# Retinopathy in newly-diagnosed systemic lupus erythematosus: should we screen for ocular involvement?

**DOI:** 10.1186/s41927-021-00203-5

**Published:** 2021-10-01

**Authors:** Hamidreza Bashiri, Nooshin Karimi, Shayan Mostafaei, Azarakhsh Baghdadi, Mohammad Nejadhosseinian, Seyedeh Tahereh Faezi

**Affiliations:** 1 Department of Internal Medicine, Shahid Sadoughi Hospital, Shahid Sadoughi University of Medical Sciences, Yazd, Iran; 2 Rheumatology Research Center, Tehran University of Medical Sciences, North Amirabad street, Tehran, 1411713137 Iran; 3 Department of Biostatistics, School of Health, Kermanshah University of Medical Sciences, Kermanshah, Iran

**Keywords:** Systemic lupus erythematosus, Retinopathy, Ocular manifestations, Vasculopathy, Disease activity

## Abstract

**Background:**

Ocular manifestations are common in systemic lupus erythematosus (SLE). Retinopathy has previously been linked to disease severity and might have a significant impact on the patient’s quality of life and has also been associated with a poor prognosis in SLE. This study aimed to determine the prevalence of retinopathy among patients who are newly diagnosed with SLE.

**Methods:**

In a cross-sectional study, patients diagnosed with SLE at a tertiary referral clinic were assessed for inclusion between March 2016 and March 2017. Patients who had received treatment for SLE at any time were excluded, as well as patients with hypertension, diabetes mellitus, and coagulopathy. Clinical findings and laboratory test results were recorded, and patients were examined by an ophthalmologist for evidence of retinal pathologies. SLE disease activity index was also calculated for all patients.

**Results:**

With 114 patients included in the final analysis, we found a prevalence of 15.8% for retinopathy among newly-diagnosed SLE patients. Cotton-wool spots were the most common finding (78%). Patients with retinopathy had significantly lower hemoglobin levels, C3 and C4 concentrations, and higher ANA and Anti-dsDNA levels. Also, patients with retinopathy had a significantly higher SLE DAI score.

**Conclusions:**

We found a relatively high rate of retinopathy in SLE patients at the time of their initial diagnosis. Our findings suggest that retinopathy is an early manifestation of the disease. Ophthalmologic screening might be considered for SLE patients at the time of diagnosis, especially for those with severe disease. We also encourage researchers to further evaluate the correlation between retinopathy and disease activity, and the prognosis of ocular involvement.

## Background

As an antibody-mediated autoimmune disease, systemic lupus erythematosus (SLE) affects virtually every organ. While cutaneous, renal, and musculoskeletal involvement is more common, ocular manifestations of SLE have also been reported in at least a third of the patient [1, 2]. Although ocular manifestations are not recognized as a diagnostic criterion by the American College of Rheumatology (ACR), their effects on the patient’s quality of life should not be overlooked, as potentially-devastating complications are not rare, and treatment becomes substantially less effective at later stages of involvement [3–7].

While data is scarce and based on low-level evidence, approximately a third of SLE patients manifest ocular involvement [1, 3, 4, 6, 8–11]. Ocular manifestations of SLE might be a primary finding related to the disease pathophysiology (e.g., retinopathy), due to a secondary disorder (e.g., Keratoconjunctivitis sicca, associated Sjögren’s syndrome (SS)), or a complication of treatment (e.g., hydroxychloroquine toxicity, drug-induced optic neuritis). More importantly, several studies have found a correlation between disease activity, either based on accepted disease activity scores or immunologic lab tests, and the ocular manifestations in SLE. Moreover, the presence and severity of ocular manifestations have been linked to poor prognosis [2, 4–7, 11, 12].

Any eye and periorbital structure might be affected by SLE. Keratoconjunctivitis sicca due to secondary SS has been reported as the most common ocular manifestation of SLE. Involvement of the eyelids, periorbital tissues, cornea, sclera, and conjunctiva have also been reported with varying frequencies [1, 9, 10, 13]. However, posterior segment involvement, i.e., retinopathy, choroidal disease, and optic neuritis, is more concerning for several reasons. First, these lesions are common, with a prevalence of 3–29%, depending on the study population and disease activity. Second, vision loss has been reported as a consequence of delayed diagnosis and treatment [14–16]. Third, as is the case with hypertension and diabetic retinopathy, patients are not symptomatic at early stages of retinal involvement, and the diagnosis could only be made if the clinician is cognizant of such pathologies. Finally, treatment of posterior segment involvement is efficacious, especially if the lesions are diagnosed early [3, 6, 7, 11, 13, 17]. Therefore, with the implications they have on the diagnosis, treatment, and prognosis of the disease, ocular manifestations of SLE merit further exploration.

The current literature on the subject is largely retrospective case series with a heterogeneous study population, and perhaps more importantly, disease duration. There is no clear evidence on how prevalent ocular disease is at the time of diagnosis or whether such involvement happens later during the disease course. If the latter is true, screening for ocular involvement might not be beneficial when SLE is diagnosed. However, if ocular lesions are prevalent in newly-diagnosed patients at a nascent pathologic stage, screening for such lesions would be warranted to ensure early diagnosis, prompt treatment, and proper follow-up.

Therefore, we designed this study to determine the prevalence of ocular involvement in newly-diagnosed SLE patients. Specifically, as retinopathy is the most common and influential posterior-segment pathology in SLE, the primary aim of this study was to determine the prevalence of retinopathy, describe the patterns of involvement, and its possible correlation with disease activity.

## Methods

After obtaining IRB-approval, a cross-sectional study was performed at a tertiary-referral SLE clinic in Tehran, Iran, from March 2016 through March 2017. Inclusion criteria included adult patients diagnosed with SLE according to the ACR criteria [18] who: 1) were newly diagnosed with SLE at our clinic, or 2) were diagnosed at an outside hospital but received no treatment and were referred for further assessment and treatment plan, or a second opinion. Patients with systemic hypertension and diabetes mellitus were excluded, due to the overlap in retinal involvement and the confounding effect it entails. Also, patients with coagulopathies and those being treated with anticoagulants were excluded for the same reason. After a description of the study protocol, goals, and implications, patients who satisfied the inclusion criteria were invited to take part in this study and signed a written consent. Next, they were visited by a fellowship-trained ophthalmologist with no prior knowledge of the clinical presentation or disease severity.

Demographic data and clinical manifestations of SLE were recorded, with an emphasis on constitutional symptoms, mucocutaneous and musculoskeletal involvement, headache, cerebrovascular involvement, neurologic symptoms, vasculitis, cardiopulmonary involvement. Laboratory tests and immunological profile were done for all patients, including a complete blood count, erythrocyte sedimentation rate (ESR), C-reactive protein (CRP), serum creatinine, liver enzymes, urinalysis, anticardiolipin antibody (aCL), anti-nuclear antibody (ANA), anti-dsDNA antibody and serum complement (C3 and C4). Disease activity was scored based on the Systemic Lupus Erythematous Disease Activity Index (SLEDAI) [19].

The ophthalmologist visiting the patient collected patients’ ocular symptoms and performed a slit-lamp examination, reporting all findings with emphasizing on retinal findings. Optic atrophy, papilledema, cotton wool spots, intraretinal hemorrhages and retinal vascular attenuation were considered retinopathy in this study. Following data collection, patients were divided in two groups based on the presence or absence of retinopathy, in order to elucidate the differences between groups.

An a priori power analysis was performed to determine the minimal sample size, utilizing G*Power v3.1. To detect a significant difference in the prevalence of retinopathy with the previous literature, using the parameters of alpha = 0.05, power = 0.80, and an effect size of 0.1, 109 patients were needed for this study.

### Statistical analysis

Data were analyzed with IBM SPSS Statistics for Windows, Version 25.0 (Armonk, NY). A Kolmogorov-Smirnov test was used to evaluate the normality of data distribution. The student’s t-test and the Mann-Whitney test were used for parametric and non-parametric variables, respectively. Chi-square/Fischer’s exact test was used for categorical data. A *P* values < 0.05 was considered significant.

## Results

In total, 114 patients newly-diagnosed with SLE were enrolled in this study, with a mean age of 31.4 ± 9.5 years (range, 19–46), of which 99 (87%) were female. None of the patients had any ophthalmic symptoms, including dryness, pain, proptosis, diplopia, decreased visual acuity, or red eye. On the slit-lamp exam, 18 patients (15.8%) were found to have retinopathy, of which 16 (89%) were female. Retinal findings included cotton-wool spots in 14 patients (78%), retinal hemorrhage in 3 patients (17%), and Roth spot in 1 (5%).

While patients with retinopathy were slightly younger, this comparison did not reach statistical significance (28.1 vs 32 years, *P* = 0.08). Patients with retinopathy had a significantly higher SLEDAI compared to those without (22 ± 7.9 vs 11 ± 6, *P* < 0.001). The prevalence of all clinical findings were statistically similar between groups (Table 1).

**Table 1:** Clinical and laboratory findings in our study population. Values are presented as mean ± SD or frequency (percent) when appropriate

Variable	All patients (***n*** = 114)	Patients with retinopathy (***n*** = 18)	Patients without retinopathy (***n*** = 96)	***P*** value
Age (years)	31.4 ± 9.5	28.1 ± 10.3	32 ± 9.2	0.08
Female gender	99 (87)	16 (89)	83 (86)	0.77
Clinical findings				
Fever	18 (16)	4 (22)	14 (15)	0.41
Headache	7 (6)	2 (11)	5 (5)	0.33
CVA	2 (2)	0 (0)	2 (2)	0.7
Psychosis/delirium	1 (1)	1 (6)	0 (0)	0.15
Seizure	21 (18)	4 (22)	17 (18)	0.65
Oral ulcers	12 (11)	4 (22)	8 (8)	0.07
Rash	66 (58)	11 (61)	55 (57)	0.76
Vasculitis	15 (13)	2 (11)	13 (13)	0.78
Arthritis	88 (77)	14 (78)	74 (77)	0.94
Myositis	3 (3)	0 (0)	3 (3)	0.44
Laboratory findings				
WBC count	6505 ± 3053	5559 ± 2971	6683 ± 3051	0.15
Hemoglobin (mg/dl)	10.8 ± 1.8	10 ± 2.1	11 ± 1.8	**0.01**
Platelet count	2.14*10^5^ ± 1.16*10^5^	2.46*10^5^ ± 1.38*10^5^	2.09*10^5^ ± 1.12*10^5^	0.24
ESR (mm/hr)	44.5 ± 27.5	53 ± 24	43 ± 28	0.14
CRP (mg/L)	13.6 ± 31.5	33 ± 70	10 ± 15	0.65
Serum creatinine (mg/dl)	0.80 ± 0.27	0.91 ± 0.31	0.79 ± 0.27	0.475
Urinalysis				
Proteinuria (mg/g)	773 ± 308	905 ± 350	749 ± 295	0.18
Casts	8 (7)	4 (22)	4 (14)	**0.006**
AST	42.1 ± 60.6	64 ± 66	38 ± 59	0.056
ALT	42.7 ± 75.8	52 ± 71	41 ± 77	0.053
Anti-Cardiolipin Ab	14.8 ± 25.0	14 ± 20	15 ± 26	0.29
ANA	4.6 ± 0.95	5.7 ± 1.1	4.5 ± 0.8	**0.01**
Anti-dsDNA	77.0 ± 22.5	109 ± 22	71 ± 17	**0.04**
Serum C3	80.2 ± 18.5	55 ± 15	85 ± 15	**0.001**
Serum C4	77.6 ± 33.2	12 ± 10	90 ± 18	**0.03**
SLE DAI	12.7 ± 7.4	22 ± 7.9	11 ± 6	**< 0.001**

Significant comparisons are indicated in bold

*CVA* cerebrovascular accident, *WBC* white blood cells, *ESR* erythrocyte sedimentation rate, *CRP* c-reactive protein, *AST* aspartate transaminase, *ALT* alanine transaminase, *ANA* anti-nuclear antibody, *SLE DAI* systemic lupus erythematous disease activity index

Patients with retinopathy were significantly more likely to have casts in their urinalysis (*P* = 0.006). Also, they had significantly lower hemoglobin levels (*P* = 0.012). Serum C3 and C4 levels were significantly lower in patients with retinopathy (55 ± 15 vs 85 ± 15, *P* = 0.001; 12 ± 10 vs 90 ± 18, *P* = 0.036, respectively). Furthermore, ANA and anti-dsDNA autoantibody levels were significantly higher in patients with retinopathy (5.7 ± 1.1 vs 4.5 ± 0.8, *P* = 0.012; 109 ± 22 vs 71 ± 17, *P* = 0.049, respectively). Other laboratory tests were not significantly different between groups. Table 1 summarizes the clinical and laboratory findings among our study population.

## Discussion

Ocular manifestations have long been reported as common findings in SLE. Although not part of the ACR diagnostic criteria, it has highest possible effect on the disease activity according to SLEDAI. While the literature is conclusive that ocular involvement is common and merits attention, it is not known whether ocular involvement is present at the time of diagnosis or develops later in the disease process. Looking to answer this question, this study is the first report of the prevalence of retinopathy in SLE at the time of diagnosis.

The most important finding of this study is that 15.8% of newly-diagnosed SLE patients had evidence of retinopathy, as examined by an ophthalmologist. This is substantially higher than previous reports on ambulatory patients, but still lower than admitted patients with an active disease, in whom a prevalence of up to 50% has been reported [1–4, 6–9, 13, 17].

Based on isolated case reports, some authors have suggested that ocular manifestations might be a presenting symptom of SLE [1, 3, 5, 13, 17, 20]. As stated previously, the literature on the subject is limited to patients already being treated for SLE, and therefore this conclusion is not backed by evidence. We would argue to the contrary of this statement, as we did not find a single ophthalmic symptom in 114 patients who were newly-diagnosed with SLE. Periorbital and anterior-segment manifestations of SLE, especially dry eye syndrome, are commonly associated with secondary pathologies including Sjogren’s syndrome. Therefore, we did not expect to find a high prevalence of such involvement. However, we believe that 16% of asymptomatic patients having evidence of retinal involvement is an alarmingly-high rate for newly-diagnosed patients and warrants a paradigm shift from a reactionary approach to ocular involvement towards incorporating ophthalmic examination as a screening test for all patients who are diagnosed with SLE. To decrease the confounding effect, we excluded patients with underlying hypertension, diabetes mellitus, and coagulopathy (either primary or drug-induced) to have a clear picture of retinal involvement in SLE. Also, we took extra care to only include patients who had never received treatment for SLE to prevent misdiagnosing medication-related retinal toxicity. However, while the stringent inclusion criteria we employed in this study decreases the likelihood of a false-positive diagnosis, it also means that the prevalence of retinopathy in the everyday clinical setting would be higher than observed in this study.

The most common findings among patients with retinopathy were cotton-wool spots, retinal hemorrhage, and Roth spots, which is in line with previous studies [3, 4, 6, 8–11, 13]. All of these pathologies reflect vascular damage. We did not find any cases of retinal vein or artery occlusion or choroidopathy, which might be due to the fact that our patients were newly-diagnosed and these lesions might be indicative of a longer duration of the disease. Although patients with retinopathy tended to be younger, clinical findings and diagnostic criteria were not significantly different between groups. However, patients with retinopathy had significantly lower hemoglobin levels. Seth et al. reported similar findings, and also reported a higher incidence of autoimmune hemolytic anemia in SLE patients with retinopathy [7]. Similar to previous reports, we found that patients with retinopathy have lower C3 and C4 concentrations, and have higher levels of ANA and Anti-dsDNA, findings which indicate an antibody-mediated retinal damage pathogenesis [1–6, 8–11, 13, 17]. Urine casts were also significantly more prevalent in patients with retinopathy.

Retinopathy has been linked to a more severe disease and poor prognosis by several authors [1–11, 13, 17, 21]. It should be noted that older studies used legacy disease activity indices, which did not score ocular involvement as a marker of disease severity [2]. However, with SLEDAI as the gold-standard disease activity index, and the fact that ‘visual disturbance’ adds 8 points to the index, this comparison is not meaningful, as any group of patients with ocular involvement will have an 8-point advantage over patients without. Quite expectedly, we found that patients with retinopathy had a significantly higher SLEDAI. However, this comparison did not hold significance when 8 points were deducted from patients with retinopathy (*P* = 0.06). Although with this unvalidated method, we are not trying to imply that ocular involvement is not related to disease activity, we would like to emphasize that with the current methodology (SLEDAI), it might not be feasible to infer the correlation between retinopathy and disease activity. Retinopathy has also been linked to a poor prognosis, which we cannot comment on owing to our study design. However, we recommend a reappraisal of evidence surrounding these two concepts: the correlation between disease activity and retinopathy, and the effect retinopathy might have on the patient’s prognosis with current treatment paradigms.

We acknowledge limitations to our study, including those inherent to the study design. While we did not include patients undergoing treatment for SLE, we did not assess the effect of treatment on ocular involvement. This is the subject of a current study at our institution. Also, the prevalence of retinopathy among patients referred to a referral SLE clinic might be different than a more general setting, which we did not have a workaround for. On the other hand, excluding patients treated for SLE and those with hypertension and diabetes, and performing an a priori power analysis are among the strengths of this study.

## Conclusions

In summary, in the study of 114 newly-diagnosed SLE patients, we found a 15.8% prevalence of retinopathy, while none of the patients had ophthalmologic symptoms. Patients with retinopathy had lower hemoglobin, C3, and C4 levels, and higher ANA and Anti-dsDNA levels. Our findings suggest that retinopathy might be an early manifestation of the disease, and ophthalmologic screening might be considered for SLE patients at the time of diagnosis, especially for those with severe disease. We also encourage researchers to further evaluate the correlation between retinopathy and disease activity, and the prognosis of ocular involvement with current treatment algorithms.

## Data Availability

The datasets used and/or analyzed during the current study are available from the corresponding author on reasonable request.

## Abbreviations

SLE: Systemic lupus erythematosus
ANA: Anti-nuclear antibody
Anti-dsDNA: Anti double-strand DNA
SLE DAI: Systemic lupus erythematosus disease activity index
ACR: American College of Rheumatology
SS: Sjogren’s syndrome
ESR: Erythrocyte sedimentation rate
CRP: C-reactive protein
aCL: Anticardiolipin antibody
